# Inhibiting MAPK14 showed anti-prolactinoma effect

**DOI:** 10.1186/s12902-020-00619-z

**Published:** 2020-09-07

**Authors:** Qiao-yan Ding, Yu Zhang, Li Ma, Yong-gang Chen, Jin-hu Wu, Hong-feng Zhang, Xiong Wang

**Affiliations:** 1 Central lab, Tongren Hospital Affiliated to Wuhan University, The Third Hospital of Wuhan, 241 Pengliuyang Road, Wuchang District, Wuhan, 430060 Hubei China; 2 Department of Pharmacy, Tongren Hospital Affiliated to Wuhan University, The Third Hospital of Wuhan, Wuhan, 430060 Hubei China; 3 College of Pharmacy, Hubei University of Chinese Medicine, Wuhan, 430065 Hubei China; 4 Department of Pathology, The Central Hospital of Wuhan, Tongji Medical College, Huazhong University of Science and Technology, Wuhan, Hubei 430014 P. R. China

**Keywords:** Prolactinoma, Prolactin, Mitogen-activated protein kinase 14, Dopamine D2 receptor

## Abstract

**Background:**

The specific underlying pathogenesis of prolactinoma has not been clarified yet, to the best of our knowledge. p38 mitogen-activated protein kinase (MAPK) signaling including p38α MAPK (MAPK14), p38β (MAPK11), p38γ (MAPK12) and p38δ (MAPK13) is associated with the development and progression of several types of cancer.

**Methods:**

Immunofluorescence analysis was performed on the prolactin (PRL) and MAPK14 expressions of pituitary gland in C57BL/6 mice and human prolactinoma specimen. In the present study, the role of MAPK14 in prolactinoma was determined using estradiol-induced mice and dopamine D2 receptor knockout (DRD2^**−/−**^) mice models in C57BL/6 wild-type (WT), MAPK14^**−/**−^ and DRD2^**−/−**^MAPK14^+/−^ mice. GH3 cells were transfected with different sets of MAPK14 small interfering RNA, which to study MAPK14 and PRL expression in GH3 cells.

**Results:**

Immunofluorescence analysis showed that PRL and MAPK14 expression were colocalized and increased in the pituitary gland of mice and human prolactinoma specimen compared with the control specimen. It was shown that PRL and MAPK14 expression was colocalized and increased significantly in the pituitary gland of estradiol-injected prolactinoma mice compared with the control mice. Knockout of MAPK14 significantly inhibited tumor overgrowth, and PRL expression was decreased in estradiol-induced mice. Furthermore, MAPK14 knockout of DRD2^**−/−**^MAPK14^+/−^ mice significantly reduced the overgrowth of pituitary gland and PRL production and secretion compared with DRD2^**−/−**^ mice. MAPK14 knockout using siRNA inhibited PRL production in GH3 cells.

**Conclusion:**

These results suggest that MAPK14 serves a promoting role in the formation of prolactinoma, and highlights the potential of MAPK14 as a potential therapeutic target in the treatment of prolactinoma.

## Background

Pituitary tumors are a type of intracranial tumor which seriously damage human health. Pituitary adenomas are categorized based on primary cell origin and type of hormone secreted. There are prolactinoma, nonfunctional adenoma, growth hormone secreting adenoma and adrenocorticotropin-secreting adenoma [1]. The morbidity rate of patients with pituitary tumors is 5/100,000, of which 50% was prolactinoma [2, 3]. The tumor cells of prolactinoma secrete excess prolactin, resulting in several symptoms such as sterility, hyperprolactinemia, amenorrhea, galactorrhea and pituitary space occupying lesion [4]. The causes of prolactinoma are unclear, but may be due to hypothalamic disorders or inherent defects of pituitary cells. At present, the dopamine agonist bromocriptine which binds to dopamine D2 receptors (D2R), is frequently used as a the first-line therapeutic for treatment of prolactinoma [2]. However, ~ 10% of patients with prolactinoma patients do not respond to bromocriptine [5]. Thus, prolactinoma are a major challenge for clinical management. Therefore, it is important to determine the pathogenesis and develop novel medical treatments for treatment of prolactinoma. MAPK is an important transmitter of signals from the cell surface to the nucleus, which includes extracellular signal-regulated kinase (ERK1/2), p38 MAPK and c-jun-N terminal kinase (JNK1/2) [6]. p38 MAPK related proteins p38α (MAPK14), p38β (MAPK11), p38γ (MAPK12) and p38δ (MAPK13) share similar protein sequences. They are activated by the double phosphorylation of TGY motif in the activation loop [7]. MAPK14 is the most abundant and best-characterized isoform, and participates in several physiological processes and diseases. In recent years, several studies have shown that MAPK14 serves a crucial role in the inflammatory response in vivo using gene knockout mice [8–10]. Additionally, the protective effects of MAPK14 inhibitor has been confirmed in animal experiments, and when used to treat colitis in clinical trials [11–14].

These results suggest that MAPK14 serves an important role in the formation and development of prolactinoma. According to the literature report [15], the model of estradiol-induced prolactinoma in mice was established. Cristina [16] and Seruggia [17] confirmed that DRD2 knockout can produce a stable and reliable prolactinoma mice model (C57BL/6), so we also used the prolactinoma model of DRD2^−/−^ mice. In the present study, estradiol-induced model and DRD2^−/−^ mice models were used to study prolactinoma development in control/WT mice and in mice lacking MAPK14. The results showed that blockade of MAPK14 expression in the mice significantly reduced tumor formation in the pituitary gland, and thus PRL production and secretion in estradiol-induced and DRD2^**−/−**^ prolactinoma. Together, these results demonstrate the critical pro-oncogenic role of MAPK14 in prolactinoma, and highlights MAPK14 was expected to be potential target for treatment of prolactinoma.

## Methods

### Animals

Female C57BL/6 mice (weighing 20 ± 5 g) were purchased from the Hubei Experimental Animal Center. DRD2^−/−^ and MAPK14^**−/−**^ female mice used in the present study have been described previously [10, 18]. DRD2^**−/−**^ and MAPK14^**−/−**^ mice were cross-bred to generate female DRD2^**−/−**^MAPK14^**+/−**^ mice. All animal experiments were approved by the Ethics Committee of Tongren Hospital Affiliated to Wuhan University, The Third Hospital of Wuhan. All the mice were housed and bred in specific pathogen-free (SPF) grade cages and provided with autoclaved food and water.

### Human pituitary gland specimens

Pituitary gland paraffin samples were collected from 27 prolactinoma patients and 21 patients with other pituitary diseases all of whom underwent surgical excision at the Central Hospital of Wuhan, Tongji Medical College, Huazhong University of Science and Technology. All samples and datas of participants were anonymized. The present study was approved by the Institutional Research Ethics Committee of the Central Hospital of Wuhan, Tongji Medical College, Huazhong University of Science and Technology.

### Animal models and grouping

On the one hand, the mice were randomly assigned to control/WT (*n* = 9), Estradiol benzoate (ES) (*n* = 9) and MAPK14^−/−^ ES (*n* = 6). A total of 20 mg/kg estradiol benzoate was injected into the abdominal cavity of mice once every 4 days and for 32 days. The prolactinoma mice model was successfully established. On the other hand, the mice were randomly assigned to WT (*n* = 6), DRD2^−/−^ (*n* = 6) and DRD2^−/−^ MAPK14^+/−^ E2 (*n* = 6). At the age of 8 months, the pituitary gland of DRD2^−/−^ mice proliferates and prolactinoma forms, which is the model mouse of prolactinoma [16].

### Sample collection

After successful modeling, all mice were anesthetized with an intraperitoneal injection of 1% pentobarbital sodium. Then, blood samples were drawn from the abdominal aorta, transferred into dried tubes containing EDTA as an anticoagulant, and centrifuged at 1000×g for 15 min. The upper plasma is collected in a clean centrifuge tube and stored at 4 °C. The pituitary gland was removed after the mice were sacrificed by cervical dislocation. The pituitary gland used for immunofluore- scence experiment were routinely formalin-fixed and paraffin-embedded. The pituitary gland used in other experiments was placed in a cryopreserved tube and stored at − 80 °C. The dead mice were sent to the laboratory animal treatment center of the hospital for unified treatment.

### Immunofluorescence staining

Paraffin specimens of pituitary glands ectioned into 5 μm pieces for immunofluorescence. Paraffin sections were dewaxed using water, and tissue sections were submerged in EDTA antigen repair buffer (pH 8.0) for antigen repair. The tissues were left to cool at room temperature and subsequently washed in PBS (pH 7.4) three times. After the sections had slightly dried, a histochemical pen was used to draw a circle around the tissue, an autofluorescence quenching agent (Wuhan Servicebio Technology Co., Ltd.) was added to the circle for 5 min, the sections were rinsed with flowing water for 10 min, and BSA was added into the circle and left to incubate at room temperature for 30 min. A primary antibody against MAPK14 (cat. l4064-l-AP; 1:1000; ProteinTech Group, Inc.) or PRL (cat. IKR0116031; 1:1000, R&D Systems) were added to the sections and incubated overnight at 4 °C. The sections were washed with PBS three times and incubated with secondary antibody at room temperature for 50 min. Subsequently, the sections were washed with PBS three times and sealed with an anti-fluorescence quenching tablet, and the sections were observed under a fluorescence microscope (NIKON ECLIPSE C1). The immunofluorescent images were analyzed using image-Pro Plus 6.0 software after microphotography.

### Elisa

The expression levels of PRL in mice plasma were detected in strict accordance with the provided instructions of ELISA kits: PRL (cat. E-EL-M0083c, Elabscience, Co., Ltd). Firstly, 100 μL standard working fluid or sample were added to the corresponding plate hole and incubate for 90 min at 37 °C. After discarding the liquid in the plate, 100 μL biotinylated antibody working fluid was immediately added to the plate hole and incubated for 60 min at 37 °C. Discard the liquid in the board and wash the board there times. Secondly, 100 μL HRP enzyme conjugate working fluid was added to each plate hole and incubated for 30 min at 37 °C, discard the liquid in the plate, and wash the plate for five times. Then, 90 μL of substrate solution was added to each plate hole and incubated for about 15 min at 37 °C. Finally, 50 μL termination fluid was added to each plate hole to terminate the reaction, and the absorbance at 450 nm was measured within 3 min using a microplate reader (Molecular Devices).

### Reverse transcription-quantitative (RT-q)PCR

Total RNA from cells or tissue was extracted using an RNA extraction kit (Tiangen Biotech Co., Ltd.). cDNA was synthesized using an RNA PCR kit according to the manufacturer’s protocol (Thermo Fisher Scientific, Inc.). The thermocycling conditions for RT-qPCR were: 95 °C for 10 min; followed by 40 cycles of 95 °C for 30 s, 55 °C for 1 min and 72 °C for 30 s; followed by 95 °C for 1 min and 55 °C for 30 s. PCR was performed using a FastStart Universal SYBR Green Master mix (Roche Diagnostics) on an Mx3000P Real-Time PCR system (Agilent Technologies, Inc.). The sequences of the mouse-primers used were: β-actin forward, GTGCTATGTTGCTCTAGACTTCG; and reverse, ATGCCACAGGATTCCATACC; and PRL forward, TCAGCCCAGAAAGG- GACACTCC; and reverse, CAGCGGAACAGATTGGCAGAGG. The sequences of the rat-primers used were: β-actin forward, CTGAGAGGGAAATCGTGCGTGAC and reverse, AGGAAGAGGATGCGGCAGTGG; MAPK14 forward, GCTGGCTCG- GCACACTGATG and reverse, GCCCACGGACCAAATATCCACTG; and PRL forward, GGTTTGGTCACAACTCCCATCCC; and reverse, TGGACAATTTGGCA- CCTCAGGAAC. PRL expression was analyzed using the 2^-ΔΔCq^ method and normalized to β-actin [19].

### Cell culture

GH3 cells derived from the rat pituitary gland were purchased from BeNa Culture Collection and were cultured in DMEM (Gibco, Thermo Fisher Scientific, Inc.) supplemented with 1% FBS (Sera Pro Instruments) at 37 °C with 5% CO_2_.

### RNA interference

In total, three different sets of small interfering (si)RNA sequences against MAPK14 were designed and synthesized by Guangzhou RiboBio Co., Ltd. The sequences of the three siRNA sequences used were: i) GGTCCCTGGAGGAA- TTCAA; ii) CCGAAGATGAACTTCGCAA; and iii) GGACCTCCTTATAGACGAA. A total of 2 × 10^5^ GH3 cells/well were seeded into a 6-well plate and transfected with the different MAPK14 siRNA separately. The cells were transfected using Lipofectamine® 2000 (Invitrogen; Thermo Fisher Scientific, Inc.) according to the manufacturer’s protocol. The RNA or protein was extracted 48 h later.

### Western blotting

Mice pituitary glands were obtained and stored at − 80 °C. The samples were placed in RIPA buffer with protease inhibitors (Roche Diagnostics) to lyse the tissue protein in the homogenizing tubes, and subsequently homogenized using tissue homogenizer(Wuhan Servicebio Technology Co., Ltd.). GH3 cells were lysed in RIPA buffer to extract proteins. The protein concentration of samples were determined using a Bradford protein assay, 20 μg proteins were loaded on a 10 or 12% SDS gel, resolved using SDS-PAGE and subsequently transferred to a nitrocellulose membrane. The membranes were blocked using 5% milk at room temperature for 1 h and then incubated with the primary antibodies at 4 °C overnight. The antibodies used were: Rabbit anti-mice PRL (cat. DF6506; 1:3000; Affinity Biosciences), rabbit anti-mice MAPK14 (cat. l4064-l-AP; 1:2000; ProteinTech Group Inc.), or mouse anti-β-actin (cat. AC004; 1:2000; Abclonal Biotech Co., Ltd.). Blots were washed three times by TBST (1 × TBS, 0.1% Tween® 20), and then incubated with secondary horseradish peroxidase-conjugated antibodies which were goat anti-mouse (cat. AS003; 1:3000; Abclonal Biotech Co., Ltd.) and goat anti-mouse (cat. AS014; 1:2000; Abclonal Biotech Co., Ltd.), and signals were visualized using enhanced chemiluminescence western blotting detection reagent (Shanghai Jiapeng Technology Co., Ltd.). Protein expression was quantified using a Fluor-S-Multi Imager (Shanghai Jiapeng Technology Co., Ltd.) and Quantity One 4.2.1 software. The band intensity was normalized to the respective β-actin band.

### Statistical analysis

Each experiment was performed three or more times and all data are expressed as the mean ± standard error of the mean. GraphPad Prism 8.0 (GraphPad Software, Inc.) version was used for all statistical analysis and graph production. Comparisons between difference groups were performed using a one-way ANOVA followed by a post hoc Tukey’s test. *P* < 0.05 was considered to indicate a statistically significant difference.

## Results

### The health status of mice

All mice had no abnormality, normal diet and drinking water, and no death. There was no significant change in the body weight of the mice, which ranged from 15 to 25 g.

### PRL and MAPK14 is upregulated and colocalized in the pituitary gland of the estradiol-injected prolactinoma mice model and in human prolactinoma specimen

**Fig. 1:**
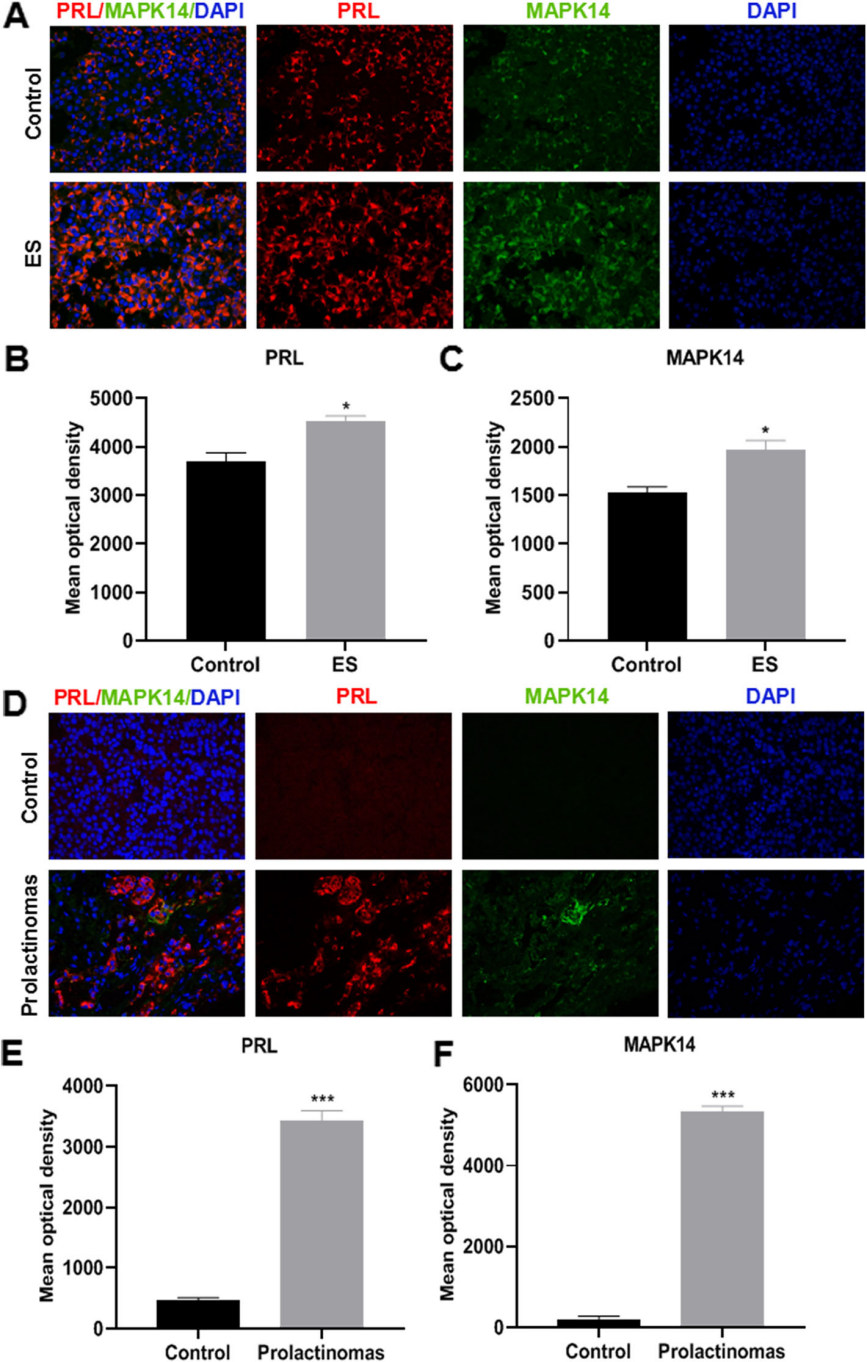
Immunofluorescence assay of PRL and MAPK14 protein expression levels. (magnification × 400). **a** PRL and MAPK14 immunofluorescence analysis of pituitary gland tissues from the control and prolactinoma model mice. **b** and **c** Mean optical density value of PRL and MAPK14 protein expression ^*^P < 0.05 vs. Control group, (*n* = 4). **d** PRL and MAPK14 immunofluorescence expression of the pituitary gland in human PRL negative specimens and human prolactinoma specimen. **e**, **f** Mean optical density value of PRL and MAPK14 protein expressions were quantitatively examined by image analysis system. ^***^P < 0.001 vs. Control group, (*n* = 4). Red PRL; Green MAPK14; Blue DAPI

Immunofluorescence analysis was performed to detect PRL and MAPK14 protein expressions levels in the pituitary gland of mice and human prolactinoma specimen. The results showed that PRL and MAPK14 expression was upregulated and colocalized in the pituitary gland of estradiol-injected prolactinoma mice (Fig. 1a-c) and human prolactinoma specimen (Fig. 1d-f). Compared with the control mice, PRL and MAPK14 expression levels in the pituitary gland of the prolactinoma mice model was significantly higher (Fig. 1b and c, *P* < 0.05). Compared with human PRL negative specimen, PRL and MAPK14 expression levels in the pituitary gland of human prolactinoma specimen was significantly higher (Fig. 1e and f, *P* < 0.001).

### MAPK14 knockout reduces the overgrowth of the pituitary gland and reduces PRL production and secretion in estradiol-injected prolactinoma mice

**Fig. 2:**
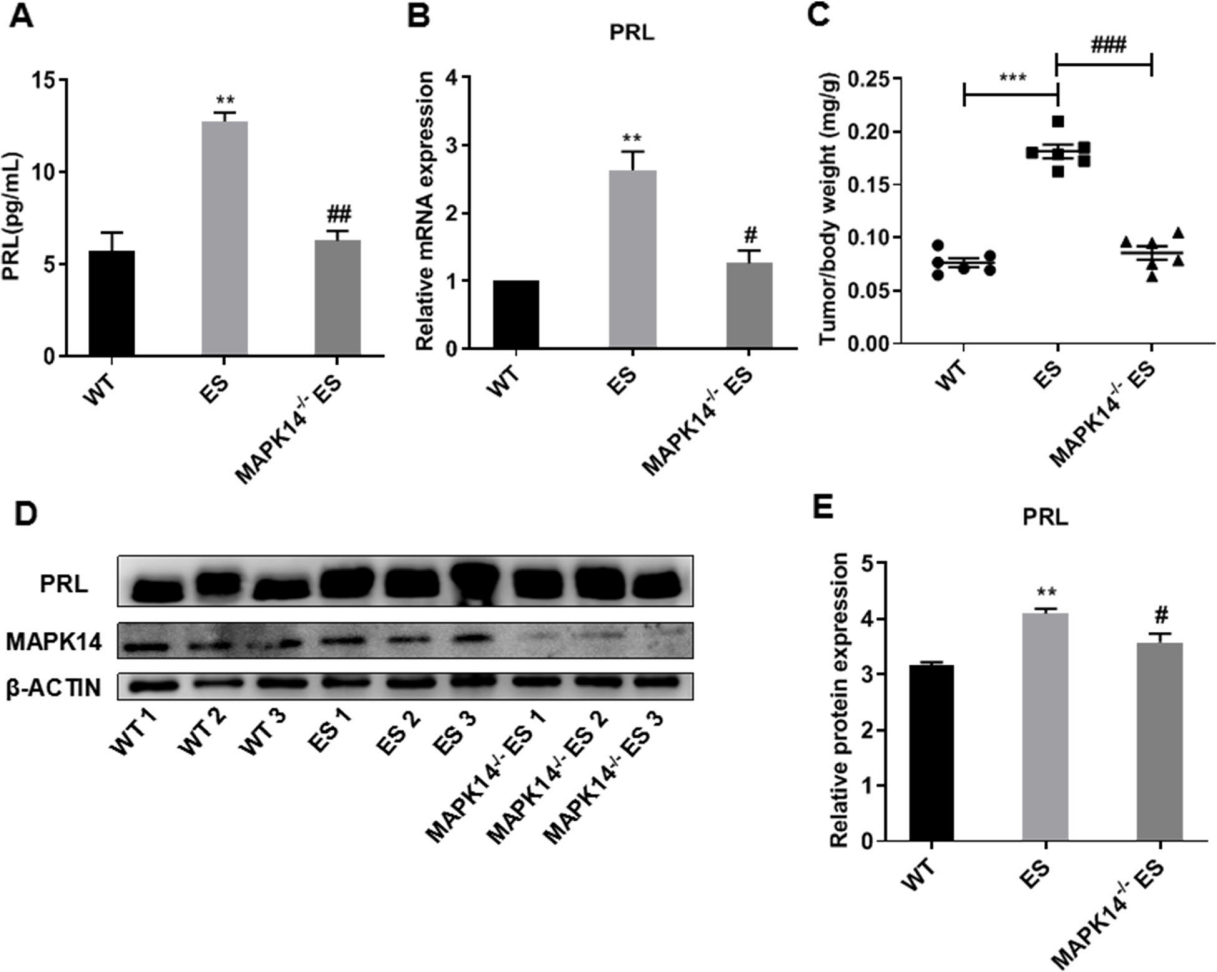
Effects of MAPK14 knockout on the development of prolactinoma in ES-induced mice. **a** Plasma PRL levels in the WT mice, ES-injected mice and ES-injected MAPK14^**−/−**^ mice. ^**^P < 0.01 vs. WT; ^**##**^*P* < 0.01 vs. E2, (*n* = 3). **b** Relative PRL mRNA expression levels in the pituitary gland of mice. ^**^P < 0.01 vs. WT; ^**##**^*P* < 0.01 vs. ES, (*n* = 3). **c** Pituitary gland weight/body weight ratio. ^***^P < 0.001 vs. WT; ^**###**^*P* < 0.001 vs. ES, (*n* = 6). **d** PRL protein expression levels in the pituitary gland of mice. **e** Relative PRL protein expression levels were quantified and normalized to β-actin. ^**^P < 0.01 vs. WT; ^**#**^*P* < 0.05 vs. ES, (*n* = 3)

Compared with the WT mice, the plasma PRL levels of peripheral blood (Fig. 2a, *P* < 0.01) and PRL mRNA expression levels of pituitary gland (Fig. 2b, *P* < 0.01) were significantly increased, and the pituitary gland weight/body weight ratio (Fig. 2c, *P* < 0.001) was also increased significantly in the estradiol-injected mice. Compared with the estradiol-injected mice, the plasma PRL levels of peripheral blood (Fig. 2a, *P* < 0.01) and PRL mRNA expression levels in the pituitary gland (Fig. 2b, *P* < 0.05) were decreased, and the tumor/body weight ratio (Fig. 2c, *P* < 0.001) was significantly decreased in the estradiol-injected MAPK14^−/−^ mice. Western blotting analysis showed that compared with the wild type mice, PRL protein expression in the pituitary gland was significantly increased in the estradiol-injected mice (*P* < 0.01), which was reversed in the estradiol-injected MAPK14^−/−^ mice (*P* < 0.05) (Fig. 2d and e).

### MAPK14 half-knockout reduces the overgrowth of the pituitary gland and PRL production and secretion in DRD2^**−/−**^ mice

**Fig. 3:**
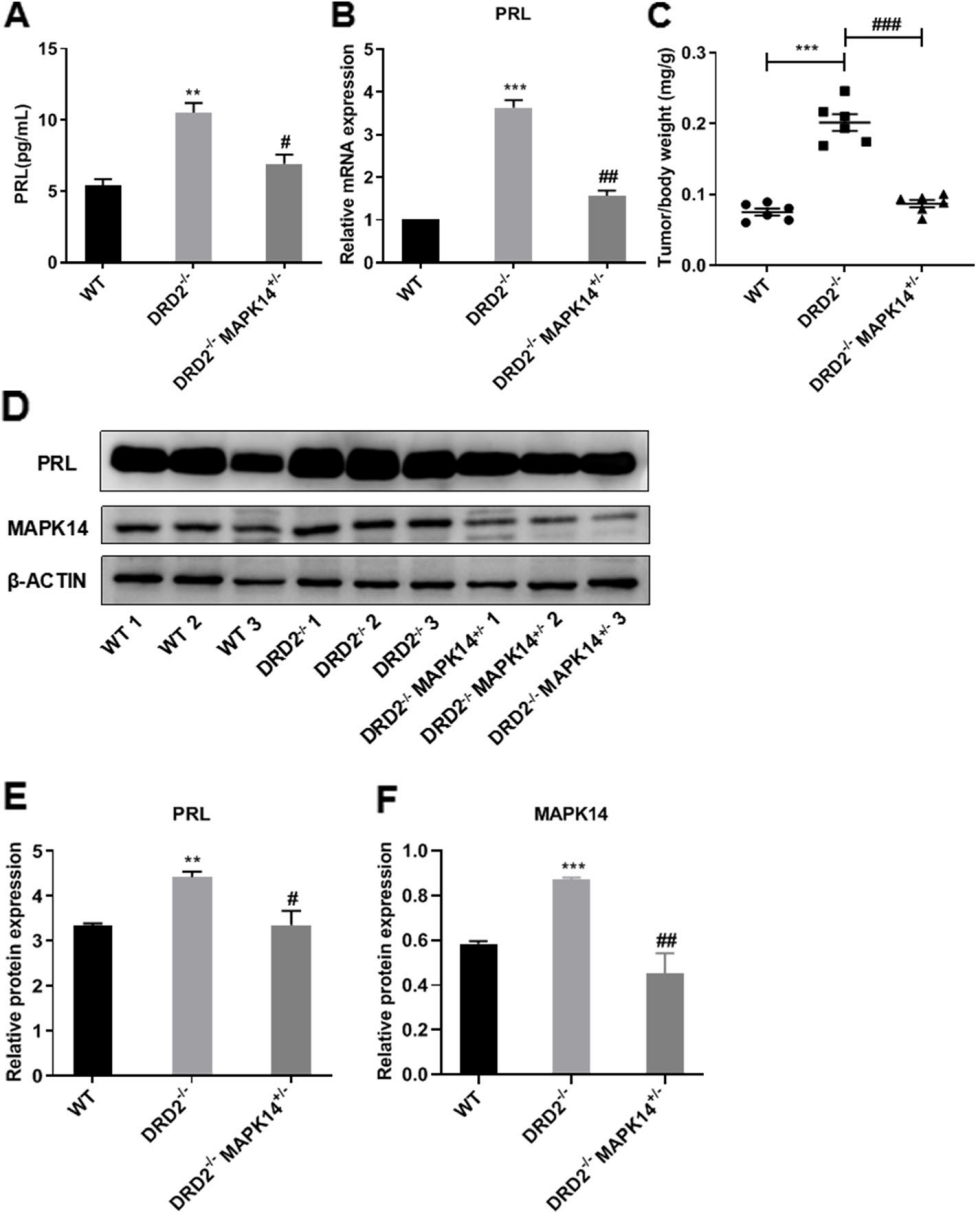
Effects of MAPK14 knockout on the development of prolactinoma in DRD2^**−/−**^ mice. **a** Plasma PRL levels in WT mice, DRD2^**−/−**^ mice and DRD2^**−/−**^MAPK14^**+/−**^ mice. ^**^P < 0.01 vs. WT mice; ^**#**^*P* < 0.05 vs. DRD2^**−/−**^ mice, (*n* = 3). **b** Relative PRL mRNA expression levels in the pituitary gland of mice. ^***^P < 0.001 vs. WT mice; ^**##**^*P* < 0.01 vs. DRD2^**−/−**^ mice, (*n* = 3). **c** Pituitary gland weight/body weight ratio. ^***^P < 0.001 vs. WT mice; ^**###**^*P* < 0.001 vs. DRD2^**−/−**^ mice, (*n* = 6). **d** PRL protein expression levels in the pituitary gland of mice. **e** Relative PRL protein expression levels were quantified and normalized to β-actin. ^**^P < 0.01 vs. WT mice, ^**#**^*P* < 0.05 vs. DRD2^**−/−**^ mice, (*n* = 3)

Compared with the WT mice, the plasma PRL levels of peripheral blood (Fig. 3a, *P* < 0.01), the PRL mRNA expression levels of the pituitary gland (Fig. 3b, *P* < 0.01), and the pituitary gland weight/body weight ratio (Fig. 3c, *P* < 0.001) were all significantly increased in the DRD2^−/−^ mice. Compared with the DRD2^−/−^ mice, the plasma PRL levels in the peripheral blood (Fig. 3a, *P* < 0.01) and PRL mRNA expression levels in the pituitary gland (Fig. 3b, *P* < 0.05) were decreased, and the tumor/body weight ratio (Fig. 3c, *P* < 0.001) was decreased significantly in the DRD2^−/−^MAPK14^+/−^ mice. Western blotting showed that compared with the wild type mice, PRL protein expression in the pituitary gland was significantly increased in the DRD2^−/−^ mice (*P* < 0.01), which was reversed in the DRD2^−/−^MAPK14^+/−^ mice (*P* < 0.05) (Fig. 3d-f).

### MAPK14 knockdown by siRNA reduces PRL production in GH3 cells

**Fig. 4:**
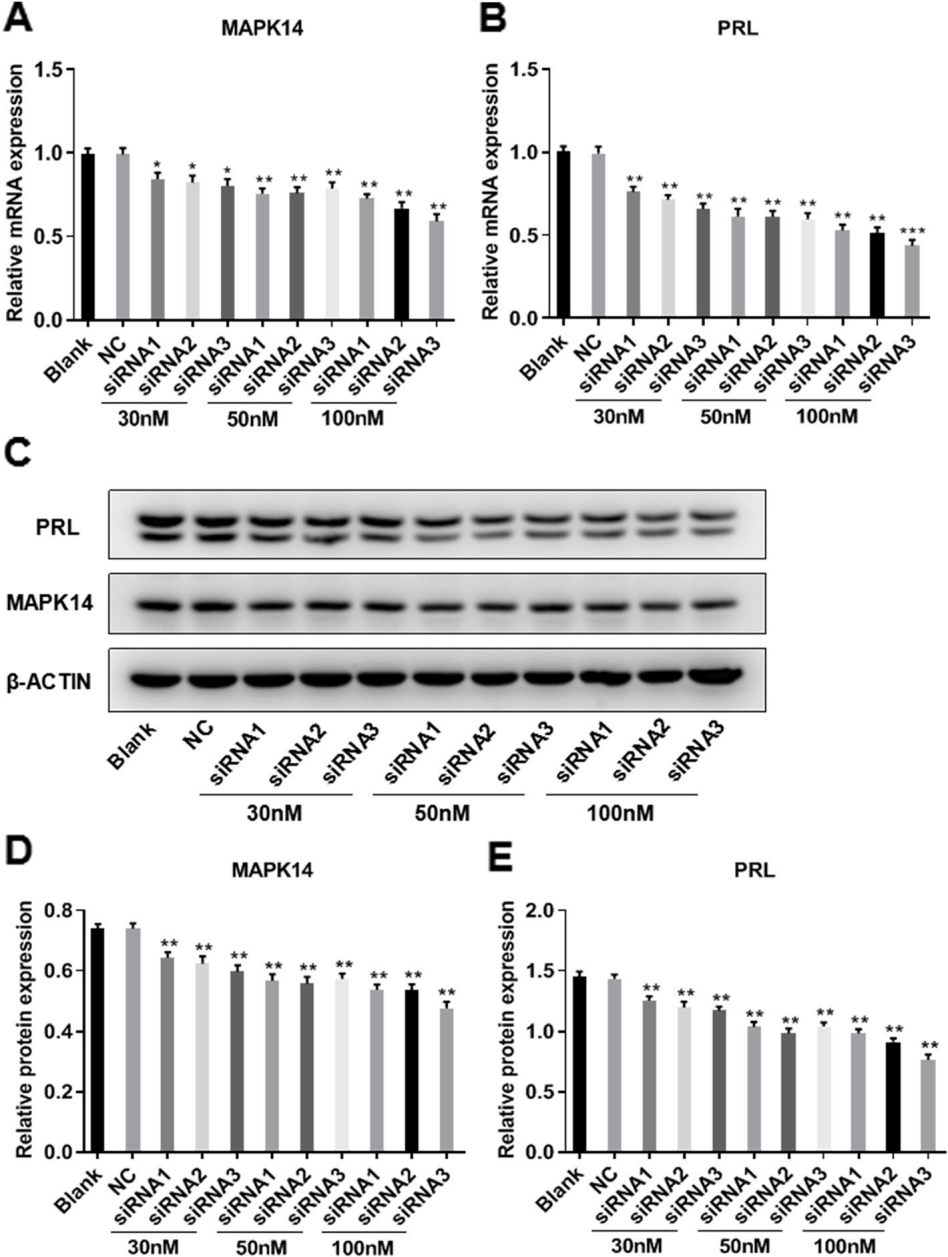
MAPK14 siRNA reduces PRL production in GH3 cells. **a** Effect of MAPK14 siRNA (30, 50 or 100 nM) on MAPK14 mRNA expression levels in GH3 cells. ^*^P < 0.05 and ^**^P < 0.01 vs. NC, (*n* = 3). **b** Effect of MAPK14 siRNA (30, 50 or 100 nM) on PRL mRNA expression levels in GH3 cells. ^**^P < 0.01 and ^***^P < 0.001 vs. NC, (*n* = 3). **c** Effect of MAPK14 siRNA (30, 50 or 100 nM) on MAPK14 and PRL protein expression levels in GH3 cells. **d** and **e** Relative protein expression of both MAPK14 and PRL were quantified and normalized to β-actin. ^**^P < 0.01 vs. NC, (*n* = 3)

GH3 cells were transfected with different sets of MAPK14 siRNA. RT-qPCR was used to determine the mRNA expression levels of MAPK14 and PRL, and protein expression was determined by western blotting. Compared with the control group, transfection of MAPK14 siRNA (30, 50 or 100 nM) significantly decreased the expression of MAPK14 in GH3 cells in a dose-dependent manner (Fig. 4a, c and d), which resulted in significantly decreased PRL expression in a dose-dependent manner (Fig. 4b, c and e).

## Discussion

The majority of patients with prolactinoma respond to dopamine agonist bromocriptine, and treatment results in the reduction of tumor size and decreased PRL production and secretion [20]. Bromocriptine, which acts on DRD2 is regarded as a first-line therapeutic for treating prolactinoma [21]. However, 10% of patients with prolactinoma exhibit severe drug resistance, even at high doses of bromocriptine, and the molecular mechanisms underlying the development of resistance have not been determined yet [22]. There are no suitable options for treatment of patients with bromocriptine resistant prolactinoma. This phenomenon highlights a significant challenge for clinical treatment. Therefore, it is important to examine the pathogenesis of prolactinoma to identify novel therapeutic targets with clinical significance.

The MAPK pathway is associated with various types of cancer [23]. There are several reports showing that p38 MAPK participates in the development of liver cancer [24], breast cancer [25, 26], lung cancer [27], bladder cancer [28, 29], prostate cancer [30–32], leukemia [33] and transformed follicular lymphoma [34, 35]. However, the relevance of the p38 MAPK14 pathway in the development of prolactinoma has not been determined yet, to the best of our knowledge. Estradiol [15] and DRD2 [36] knockout can induce the formation of prolactinoma in mice. In the present study, two methods were used to successfully construct a mouse model of a prolactinoma [36], and MAPK14 was knocked out to determine its role in the development of prolactinoma in mice. The results showed that PRL and MAPK14 protein expression was colocalized and expression was significantly higher compared with the control group in both the estradiol-injected prolactinoma mice and human prolactinoma specimen, suggesting that MAPK14 was significantly associated with PRL expression. In vivo, the absence of MAPK14 significantly reduced the growth of prolactinoma in estradiol-injected mice and DRD2^−/−^ mice, and reduced the production and secretion of PRL. In vitro experiments have shown that inhibition of MAPK14 can significantly reduce the expression of PRL in GH3 cells. Together, these results indicate that MAPK14 serves an important role in promoting the development and progression of prolactinoma in mice.

The MAPK family is a group of serine/threonine protein kinases that are very well-conserved in eukaryotes. They are a major group of signaling molecules involved in signal transduction, and serve important roles in the development and progression of diseases, and are involved in a variety of cellular processes, such as proliferation, differentiation, transcriptional regulation and development [37]. Substrates of MAPK include transcription regulators (such as, ATF2, MEF2C and MAX) and cell cycle regulators (such as, CDC25B and tumor suppressor p53). MAPK serves an important role in stress-related transcription and cell cycle regulation [38]. MAPK14 is an important member of p38 MAPK, which is typically highly expressed in the majority of cell types and can be activated by physiological stress, lipopolysaccharide (LPS), osmotic stress and ultraviolet irradiation [39]. LPS, tumor necrosis factor, platelet activating factor interleukin-1 and inflammatory stimuli, such as ischemia/reperfusion can induce the activation of intracellular p38 MAPK, thereby promoting cell proliferation and inhibiting apoptosis [40].

PRL is a polypeptide hormone synthesized and secreted by lactotroph cells in the anterior pituitary gland and involved in a wide range of functions beyond reproduction and lactation, including metabolic behavioral immune regulation and osmotic regulation [41]. However, overexpression of prolactin results in endocrine disorders, which affects normal reproductive function [42]. The release of pituitary prolactin is primarily inhibited by the neurohormone dopamine [43]. Dopamine directly activates the D2 receptor on the cell membranes of lactotrophs of the anterior pituitary, reducing the production and secretion of PRL [44]. In the present study, DRD2 knockout mice were selected as the model, and inhibition of MAPK14 reduced the secretion of PRL and growth of tumor, suggesting that DRD2 activation is not the only means of regulation of expression of PRL. In future studies, the specific molecular mechanisms of MAPK14-mediated regulation in the production and secretion of PRL should be examined.

In summary, the present study elucidates the role of MAPK14 in promoting tumor growth, and the production and secretion of PRL through in vitro and in vivo experiments. The role of MAPK14 in promoting the development of prolactinoma highlights novel avenues for the study of the pathogenesis of prolactinoma. Inhibition of MAPK14 expression may be a novel therapeutic target for the treatment of prolactinoma.

## Conclusion

This was the first study to demonstrate that inhibition of MAPK14 can reduces the growth of prolactinoma, and the production and secretion of PRL. This confirms that inhibition of MAPK14 showed anti-prolactinoma effects.

## Data Availability

All data generated or analyzed during this study and supporting our findings are included and can be found in the manuscript. The raw data can be provided by corresponding author on reasonable request.

## Abbreviations

PRL: Prolactin
MAPK: Mitogen-activated protein kinase
DRD2: Dopamine D2 receptor
siRNA: Small interfering RNA
ELISA: Enzyme-Linked Immuno sorbent Assay
ES: Estradiol benzoate
WT: Wild type
